# The butyrate-producing Gram-positive human gut bacterium, *Hoskinsella mucinilytica*, selectively targets host mucin *N*-acetylhexosamines

**DOI:** 10.1016/j.jbc.2026.111371

**Published:** 2026-03-17

**Authors:** Nicholas A. Pudlo, Gabriel Vasconcelos Pereira, Sadie R. Schaus, Qinnan Yang, Jaime J. Fuentes, Chunsheng Jin, Robert Hein, Li Zhang, Nicolas Terrapon, Costas A. Lyssiotis, Thomas M. Schmidt, Gunnar C. Hansson, Ana S. Luis, Eric C. Martens

**Affiliations:** 1 Department of Microbiology and Immunology, University of Michigan, Ann Arbor, Michigan, USA; 2 Department of Medical Biochemistry and Cell Biology, Institute for Biomedicine, Sahlgrenska Academy, University of Gothenburg; 3 Department of Internal Medicine, University of Michigan, Ann Arbor, Michigan, USA; 4 Department of Molecular & Integrative Physiology, University of Michigan, Ann Arbor, Michigan, USA; 5 Aix Marseille Univ, CNRS, UMR7257 AFMB, Marseille, France; 6 INRAE, USC1408 AFMB, Marseille, France; 7 Department of Ecology and Evolutionary Biology, University of Michigan, Ann Arbor, Michigan, USA; 8 SciLifeLab, University of Gothenburg, Gothenburg, Sweden

**Keywords:** feces, microbiome, mucin, carbohydrate metabolism, glycoside hydrolase, glycoprotein, bacterial transcription, proteomics, fatty acid

## Abstract

The personalized microbial communities (micobiota) that inhabit the distal guts of humans have evolved to process a variety of complex carbohydrates. Many gut bacteria depolymerize and ferment dietary fiber polysaccharides, mutualistically providing short-chain fatty acids and other metabolites to their host. Some human gut bacteria have evolved to utilize components of host mucin glycoproteins—the major component of secreted mucus that protects the gut. Recent studies have implicated certain mucin-degrading bacteria in the development of intestinal inflammation, making the identification of new gut bacteria that possess the ability to degrade mucins an important goal. We used gastric mucin to isolate a novel bacterium, *Hoskinsella mucinilytica*, that is largely restricted to using the GlcNAc and GalNAc sugars found in the *O*-linked glycans appended to secreted mucin. This butyrate-producing bacterium accesses these sugars from both polymeric gastric mucin and chemically released oligosaccharides. It also has a genome with correspondingly restricted carbohydrate-active enzyme content, with only three demonstrated mucin-degrading glycoside hydrolase enzymes belonging to families GH31, 36, and 89. Surprisingly, strains with an identical 16S rRNA V4 region to this isolate appear to be rare in the now numerous sequence-based microbiota surveys, with only 30 of 7390 (0.41%) human subjects harboring detectable levels of this bacterium in their stool, with an overall relative abundance ranging from 0.0004% to 0.013% when it is detected. This combination of low prevalence and low abundance suggests that this species could occupy an unknown niche for which access to mucin is important but otherwise renders it difficult to detect in stool-based microbiota surveys.

The mammalian gastrointestinal (GI) tract is home to a dynamic microbial ecosystem. A variety of host and environmental features contribute to how the members of each person’s resident gut microbiota maintain colonization and coexist (1, 2, 3, 4, 5, 6, 7, 8). Complex carbohydrate acquisition is a prominent determinant of bacterial proliferation and persistence in the colon since GI contents are depleted of nutrients that the host degrades/absorbs in the small intestine (simple sugars, soluble starch, fats, and proteins) and enriched in dietary fiber polysaccharides that transit the upper GI undigested by host enzymes (9, 10, 11). Consequently, many gut bacteria have evolved to be proficient degraders of dietary fiber, a nutrient category that encompasses many chemically distinct polysaccharides. Because of the breadth and chemical diversity of fiber polysaccharides, individual gut bacteria do not degrade all fibers, but both generalists (*i.e*., degrade many fibers) and specialists (*i.e*., degrade one or a few) have been identified (12). In addition to the presence of fiber, the host continuously secretes protective mucus and sheds/replaces epithelial cells, and these reservoirs are rich in glycoconjugates like mucins (13). Secreted mucin glycoproteins, of which mucin 2 (MUC2) is the most prominent in the human and mouse colon, are potentially very rich nutritional sources since they are composed of up to 80% *O*-linked glycans, oligosaccharides that are so named because they are attached to serine and threonine residues that are abundant in mucin polypeptides. *O*-glycans incorporate up to five different monosaccharides, galactose (Gal), GalNAc, GlcNAc, fucose (Fuc), and *N*-acetylneuraminic acid (NeuNAc), plus sulfate in a variety of positions. Typically, over 10^2^ different *O*-glycan structures are present in mucin, and some terminal modifications (Fuc, NeuNAc, sulfate, and α-GlcNAc) vary along the length of the GI and between mammalian species, whereas others, like blood groups (A, B, and H), are genetically determined and therefore present only in some individuals (14, 15). Growth on mucin or its components is challenging because of the glycobiological complexity of *O*-glycans, which potentially necessitates many enzymes with different specificities to significantly degrade these structures, and the sterically hindered nature of *O*-glycans attached to MUC2 glycoprotein. Nevertheless, the constant presence of secreted mucus in the GI tract makes it a potentially stable source of nutrients, and mucin/*O*-glycans are among the few classes of complex carbohydrates for which absolute metabolic specialization (*i.e*., species only grow on mucin) has been observed among gut bacteria surveyed to date (12, 16, 17).

In the colon, the mucus layer safeguards epithelial cells and consists of a firm inner layer that is often but not always devoid of microbial colonization and a looser outer layer that overlies the firm inner mucus and is thought to travel with and encapsulate a forming fecal mass (18, 19). This outer layer is heavily colonized by bacteria, at least some of which directly degrade mucin and other host glycans and turn on the corresponding genes in this niche (20). In addition to mucin secreted by colonic goblet cells, the GI lumen also contains mucins shed from more proximal sites (salivary, gastric, and small intestine) that provide additional resources and also introduce glycobiological complexity because of *O*-glycan variations in different gut regions. The individual human histo-blood group ABO(H)–Lewis systems are reflected in the glycans coating the intestine. Interestingly, the *N*-glycans of the human small intestine also carry numerous blood group epitopes and are devoid of terminal sialic acids (21). Considering how the histo-blood groups are reflected in the intestine suggested that there should be hydrolase specificity, something that was observed early on by Cromwell and Hoskins (22) and Hoskins *et al.* (22). More recent observations have correlated host blood group *ABO* and *FUT2* genotypes with corresponding genomic variations in human gut bacteria that allow them to utilize the blood group(s) the host presents (23, 24). Still, other mucin-degrading bacteria, like *Bacteroides thetaiotaomicron*, produce enzymes that enable it to address each of the different histo-blood groups it might find, sometimes from the same gene cluster (24).

Several human gut bacteria from different phyla have been shown to degrade components of GI mucins. These include species/strains belonging to *Bacteroides* and *Parabacteroides*, *Ruminococcus torques* and *gnavus*, *Clostridium* spp., *Bifidobacterium* spp., *Peptostreptococcus* spp., and *Akkermansia muciniphila* (25, 26, 27, 28, 29, 30, 31, 32, 33, 34, 35, 36, 37, 38, 39, 40, 41, 42, 43, 44, 45). Some of these bacteria have been implicated in positive and negative health outcomes, such as increased risk of developing inflammatory bowel diseases (IBDs) that is associated with increased abundance of *R. torques* (46, 47) and improved metabolic health in the case of *A. muciniphila* (48, 49). Whether the abilities of these strains to utilize mucin as a nutrient source relate to their effects on health remains to be determined.

To liberate sugars from glycans, bacteria must depolymerize them using appropriate carbohydrate-active enzymes (CAZymes), and typically, the number of enzymes required increases with substrate complexity (17, 50). In addition, for MUC2 and *O*-glycan degradation, which are heavily modified with sulfate in the colon, bacterial sulfatases may be necessary, although not all proficient mucin degraders possess detectable sulfatase activity (16). Sulfatases are classified in the database SulfAtlas ((51); https://sulfatlas.sb-roscoff.fr/), whereas CAZymes have been subdivided into classes, families, and subfamilies by the CAZy project ((52); http://www.cazy.org/). Degradative CAZyme classes include polysaccharide lyases and glycoside hydrolases (GHs), often associated with nonenzymatic carbohydrate-binding modules (CBMs), as well as carbohydrate esterases (CEs). GH families use hydrolytic mechanisms, and some display characteristic-level specificities toward linkages common in host *O*- and *N*-linked glycans, such as GH33 and GH29/GH95 for the release of NeuNAc and Fuc, respectively (29, 53). In contrast, multifunctional families can be divided into more specific subfamilies, such as in GH2, distinguishing between enzymes that release glucuronic acid from those that release Gal (54). Based on their evolutionary histories and forces like lateral gene transfer, the genomes of individual bacterial species often encode a suite of different CAZymes. However, only a subset of the enzymes in a given genome might be involved in targeting a particular substrate, and individual bacteria often target multiple polysaccharides, requiring empirical approaches to connect genomic potential with actual metabolic phenotypes. Making this task easier in some cases, gut bacteria often group CAZymes and other functions involved in the same metabolic process into gene clusters, and these loci are often highly expressed in the presence of their cognate substrate. Thus, functional genomics approaches like RNA-Seq are often successful in identifying candidate CAZymes involved in degrading a particular substrate, although transcriptional regulation is not always observed (16).

The recent resurgence of culturing, including enrichment culture (55, 56) to target novel or low-abundance microbes, coupled with ease of genome sequencing, has opened up new paths to understand the diverse bacteria that live in the human gut (57, 58, 59). Here, we describe the carbohydrate utilization abilities of *Hoskinsella mucinilytica* (*Hm*), a recently isolated and described species from the human gut microbiota (60). While this species is capable of degrading host mucin and the associated *O*-glycans, it is restricted to using mucin and corresponding *O*-glycans of porcine gastric origin and exhibits a strong preference for α-linked *N*-acetylhexosamine sugars, GalNAc and GlcNAc. Combined genomic, transcriptomic, and proteomic analyses provide insight into the mechanisms involved, revealing a limited set of GHs that cleave *N*-acetylhexosamines from *O*-glycans. The restricted specificity that *Hm* displays for foraging just two component sugars from intact mucin reveals yet another nuance in the relationship between symbiotic gut bacteria and the GI mucin system, namely that an organism can potentially become specialized for a particular mucin subtype and also for a subset of sugars. By removing terminal *N*-acetylhexosamines from mucin, *Hm* may have the capacity to alter the structure of the mucin it leaves behind, possibly influencing which bacteria can use these altered substrates and therefore impacting community interactions more broadly.

## Results

### *Hm* displays limited glycan use and mucin source specificity

As part of a campaign to identify novel gut bacteria capable of using various complex carbohydrates, we performed isolation on agar-solidified medium containing unmodified porcine gastric mucin (PGM) as the main carbon source added to a semidefined medium (56). Among 198 well-defined, isolated colonies derived from 83 different healthy adult fecal samples and analyzed by 16S rRNA gene sequencing, we isolated one bacterium that we recently described as a member of a novel species in the reclassified genus *Hoskinsella* (60). This species is named *Hm* (herein referred to as *Hm*; =UGRO018_0423^T^ = DSM 116809^T^ = ATCC TSD-466^T^). *Hm* is a Gram-positive, obligate anaerobe in the Bacillota phylum (formerly, Firmicutes), Erysipelotrichaceae family, for which some members have been associated with GI diseases (61). However, phenotypic studies for members of this family from the human gut remain sparse and could illuminate new roles for these microbes. To address this gap, we performed experiments to measure the carbohydrate utilization capabilities of *Hm* on a panel of 59 different mono-, oligo-, and polysaccharides that span the numerous sugars and linkages relevant to human diets and also includes microbial sources and purified mucin substrates (12, 62).

**Figure 1:**
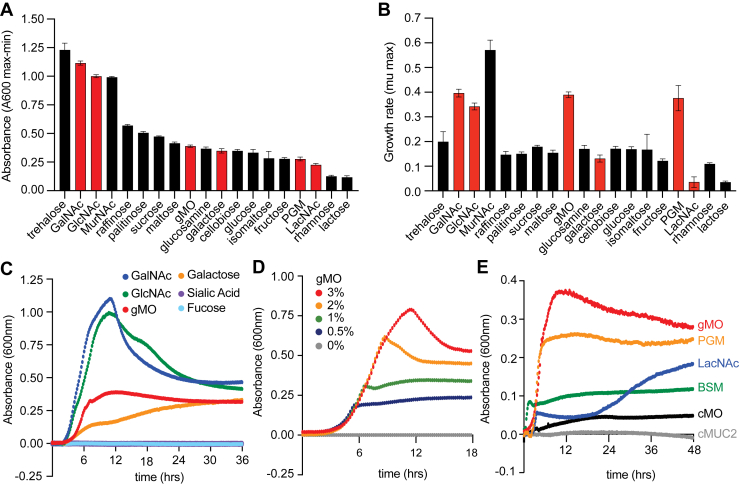
**Growth measurements reveal gastric mucin specificity by *Hoskinsella mucinilytica* (*Hm*).***A*, bar graph showing the carbon sources that *Hm* exhibits a total growth of ≥0.1 absorbance at 600 nm when normalized to a water control and starting absorbance on each carbon source before growth. Error bars indicate the standard deviation of the mean (SDM) of n = 6, except LacNAc (n = 3). GalNAc, GlcNAc, *N*-acetylmuramic acid (MurNAc), gastric mucin oligosaccharide (gMO), porcine gastric mucin (PGM), and *N*-acetyllactosamine (LacNAc). *Red bars* indicate mucin glycans or mono- or disaccharides found in mucins. *B*, bar graph showing growth rate measurements for carbon sources that *Hm* has a total growth of ≥0.1 as measured in *A*. Error bars indicate the SDM for n = 6, except LacNAc (n = 3). *Red bars* indicate mucin glycans or mono- or disaccharides found in mucins. *C*, growth curves of *Hm* on PGM *O*-linked glycans (gMOs) and mucin monosaccharides normalized to a water control (each curve represents the mean of n = 6). *D*, growth curves by *Hm* on increasing concentrations of gMO (each curve represents the mean of n = 6). *E*, growth curves of *Hm* on different mucin sources. Each curve represents the mean for multiple replicates: PGM, gMO, and bovine submaxillary mucin (BSM) were n = 6, whereas LacNAc, colonic mucin oligosaccharides (cMOs), and colonic mucin (cMUC2) were n = 3.

The ability to grow on a particular substrate (considered positive if a culture increased ≥0.1 absorbance at 600 nm from its own baseline) was limited to 32% (19/59) of the carbohydrates tested (Figs. 1, *A* and *B*, S1, and Table S1). Of the substrates considered positive for growth, 17 are either mono-, di-, or trisaccharides, revealing that *Hm* has a limited ability to degrade longer polysaccharides. In addition, many substrates that were scored as positive for growth had low growth rates, indicating that they are suboptimal substrates (Figs. 1*B*, S1, and Table S1). Consistent with *Hm* being adept at degrading and utilizing mucin glycan components, whole PGM and PGM-derived gastric mucin *O*-glycans (gMOs), as well as GalNAc and GlcNAc (two of the five monosaccharides composing mucin), were four of the five substrates that enabled the most rapid growth rates, although total growth yield for PGM and gMO was noticeably lower (*red bars* in Fig. 1, *A* and *B*). Strong growth was also observed on trehalose and the peptidoglycan sugar *N*-acetylmuramic acid. Only weak growth was observed on the disaccharide *N*-acetyllactosamine (LacNAc; [Gal-β1,4-GlcNAc]), which is a common repeat component in *O*-glycans. These growth phenotypes suggest that *Hm* is proficient at metabolizing *N*-acetylhexosamine sugars that are common in the gut environment from host mucin secretion and bacterial cell turnover.

It is notable that *Hm* is unable to use the mucin monosaccharides, Fuc and NeuNAc, and grows poorly on Gal compared with GalNAc or GlcNAc (Fig. 1*C*). To test the hypothesis that the relatively small growth yield that occurs on gMO, albeit at a rapid rate similar to GlcNAc and GalNAc (Fig. 1*B*), is potentially limited by access to GalNAc–GlcNAc sugars within the *O*-glycan chains, we grew *Hm* on increasing concentrations of gMO. In support of our hypothesis, the total growth yield increased as a function of gMO concentration (Fig. 1*D*), suggesting that *Hm* is limited by the amount of carbohydrate it can remove from gMO and may be unable to cleave additional sugars/linkages to access more GalNAc–GlcNAc. We observed poor growth on intact colonic MUC2 (cMUC2) and corresponding purified colonic mucin oligosaccharides (cMO) (27) from porcine colon (Fig. 1*E*). Both substrates contain *O*-glycans with more abundant nonreducing end NeuNAc and sulfate, which might limit access by *Hm* enzymes and therefore prevent growth. *Hm* also grew poorly on bovine submaxillary mucin, which contains less complex *O*-glycosylation and predominant *O*-linked αGalNAc (T antigen) and sialyl-T antigen (63) (Fig. 1*E*).

While some growth was observed on LacNAc, the comparatively weak growth rate and low total biomass yield suggest that this substrate is either poor at inducing potential enzymes required for its cleavage or *Hm* lacks β-galactosidase activity required to liberate GlcNAc that otherwise promotes strong growth as a free monosaccharide. Taken together, our growth analysis reveals that *Hm* is optimized to target gastric mucins (PGM) and their associated glycans (gMO). It is important to note that although PGM is utilized in this study, α-linked GalNAc and GlcNAc are also common terminal sugars found in human gastric mucins (64), suggesting that *Hm* may also access these linkages in humans from gastric mucin that arrives in the colon from mucus secreted in the stomach.

### Transcriptomic and proteomic analysis reveals a restricted subset of potential mucin-degrading CAZymes

PGM and gMO each contain >10^2^ different *O*-glycan structures that vary mostly in linkage composition and content of the five mucin monosaccharides and sulfate decorations. Since gMO is directly purified from whole PGM, there is likely an extensive overlap in their glycan composition, and *Hm* is restricted in its ability to utilize all five mucin sugars. Thus, it is conceivable that *Hm* is only equipped with enzymes to liberate GalNAc and GlcNAc from gMO and not Fuc, Gal, and NeuNAc, which do not support strong growth. However, examples exist of bacteria that remove common capping sugars from glycans’ nonreducing ends but cannot use them directly, which is the case for *Bacteroides thetaiotaomicron* cleavage of NeuNAc (65) and *R. torques* cleavage of Fuc (16), potentially making these sugars available for other species. To gain more insight into the mucin-degrading mechanisms deployed by *Hm*, we sequenced the genome of strain UGRO018_0423. The genome was determined to have a size of 3.73 Mb and encode a total of 3466 predicted protein-coding sequences, given the annotation criteria utilized. *Hm* is predicted to encode 122 total CAZymes, including 74 GHs, 39 glycosyltransferases, two polysaccharide lyases, and seven CEs. The idea that *Hm* cannot liberate Fuc and NeuNAc from *O*-glycans is supported by its genome’s CAZyme inventory, which notably does not contain bacterial GH families known to target these terminal sugars (α-fucosidases: GH29, GH95, GH139, GH141, or GH151; sialidases: GH33, GH34, GH156, GH177, or GH181). Surprisingly, approximately 40% (30/74) of the GHs encoded in the *Hm* belong to the GH1 family, which have a multitude of characterized enzymatic activities but few toward mucin glycans. This high abundance of GH1 enzymes might be related to the di- and trisaccharide utilization abilities that we observed. In contrast, only 11% (8/74) of the predicted GHs belong to families that have been previously shown to be involved in degrading mucin sugars: GH2, GH31, GH35, GH36 (three), GH89, and GH101 (Table S2) (17, 43). Finally, the SulfAtlas database only predicts two sulfatases, which could also limit the ability of *Hm* to fully depolymerize mucin substrates, especially cMO, since sulfated sugars or the absence of appropriate sulfatases are known to impede access to the underlying glycosidic linkages (27). The first sulfatase, UGRO018_02025, is assigned to the S1_64 sulfatase family, which has no known activities. However, the second sulfatase, UGRO018_02081, is predicted to be an S1_46 family sulfatase that has been shown to have exo-*N*-acetyl-d-glucosamine-3-sulfate 3-*O*-sulfohydrolase activity (27, 66).

We next sought to determine what genes and corresponding functions might be expressed during growth in the presence of gMO glycans. Thus, we performed RNA-Seq on mRNA purified from cells grown on gMO, the two *O*-glycan monosaccharides (GlcNAc and GalNAc) that support strong *Hm* growth and cellobiose, a β1,4-linked glucose disaccharide that does not contain any sugars or linkages in common with gMO. Genes were considered differentially expressed in all RNA-Seq comparisons if they exhibited ≥5-fold expression change and a *p* value ≤0.05. Since Gmo-degrading bacteria like *Bacteroides thetaiotaomicron* exhibit increased expression of several dozen genes in response to gMO compared with a simple sugar (glucose) reference (25), we hypothesized that this RNA-Seq comparison would reveal CAZymes and other functions involved in degrading gMOs. We first compared the responses during growth in gMO compared with GlcNAc or GalNAc. Interestingly, the gMO–GlcNAc comparison revealed only 89 genes that were differentially expressed: 23 genes (26%) upregulated and 66 genes (74%) downregulated (Fig. S2, *A* and *B* and Table S2). Surprisingly, no degradative CAZymes displayed significantly increased expression, which led us to consider that either GlcNAc alone (a prominent *O*-glycan component) may be sufficient for inducing genes involved in gMO degradation or the genes involved are constitutively expressed.

In contrast to the gMO–GlcNAc comparison, there were 535 genes that were differentially expressed during growth in gMO compared with GalNAc: 281 (53%) upregulated and 254 (47%) downregulated (Fig. S2, *A* and *B* and Table S3). This increased number of differentially expressed genes during growth in gMO compared with GalNAc reveals that *Hm* responds substantially different to the two mucin *N*-acetylhexosamines on which it grows. Despite the larger number of differentially regulated genes in the gMO–GalNAc comparison, only one gene encoding a GH showed significantly increased expression (∼50-fold) in gMO compared with GalNAc. This gene is annotated to encode a putative GH4 family enzyme (UGRO018_00760) whose closest characterized homologs are 6-phospho-α-glucosidases (four characterized members with 68–72% identity). GH4 family members have not been directly associated with *O*-glycan degradation, but some are α-galactosidases, which could possibly target Gal found in blood group B (terminal α-1,3-linked Gal). Although the expression difference was not significant (*p* = 0.0872) in the gMO-GlcNAc comparison, UGRO018_00760^GH4^ was also induced ∼13-fold in this comparison. However, a recombinantly expressed version of the GH4 showed no detectable activity on gMO or commercially available substrates, including those that present terminal blood group A or B epitopes (Fig. S2, *C* and *D*). In addition, no activity was detected on any *p*-nitrophenyl (*p*NP) α-linked (glucose, phosphoglucose, and Gal) and β-linked (glucose, Gal, and GlcNAc) substrates tested (data not shown). Direct comparison of gene expression between growth in GlcNAc and GalNAc revealed 488 genes that are differentially expressed: 195 (40%) expressed more highly in GlcNAc and 293 (60%) expressed more highly in GalNAc. There were only three GH-encoding genes upregulated in GlcNAc when compared with GalNAc, but all are from the GH1 family (Fig. S2, *A* and *B* and Table S4).

**Figure 2:**
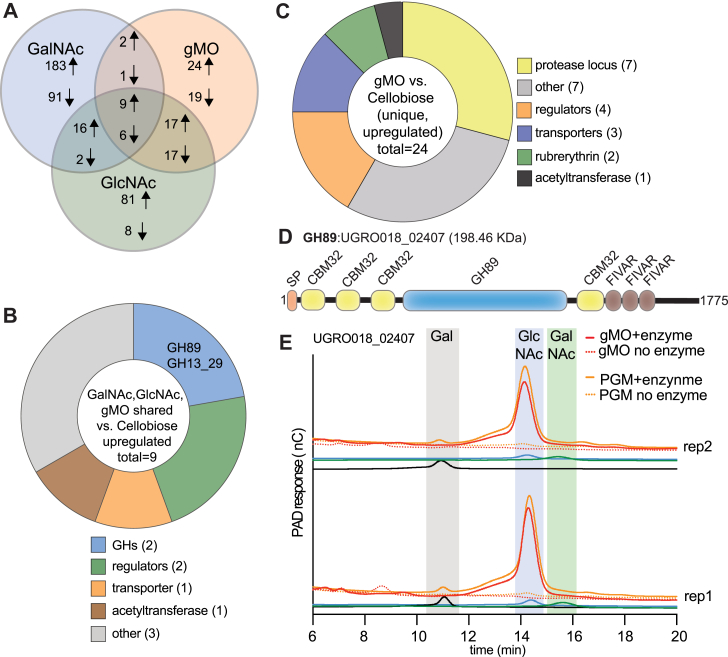
**RNA-Seq reveals a limited suite of gMO-responsive genes.***A*, Venn diagram showing significantly up- and downregulated genes in *Hm* during growth in GalNAc, GlcNAc, and gMO compared with cellobiose. Significance thresholds include having a fold change ≥5-fold with a *p* value ≤0.05. *B*, annotation categories (determined by BLAST) for shared upregulated genes between GalNAc, GlcNAc, and gMO when compared with cellobiose. These are the nine upregulated genes in the middle sector of *A*. *C*, annotation categories (determined by BLAST) for genes unique to gMO when compared with cellobiose. These are the 24 upregulated genes in the *top right sector* of *A*. *D*, protein domain structure of the gMO-responsive UGRO018_02407^GH89^ from NCBI BLASTp and EMBL-EBI InterProScan. Domain abbreviations are SP, signal peptide; CBM32, carbohydrate-binding module 32; and FIVAR, found in various architectures. *E*, HPAEC-PAD chromatograms after overnight incubation of the GH89 with gMO or PGM. *Solid lines* indicate the addition of the UGRO018_02407^GH89^ enzyme as compared with the *dotted lines*, which are controls lacking the enzyme. Standards for galactose (*black*), GlcNAc (*blue*), and GalNAc (*green*) were run at 10 μM. EBI, European Molecular Biology Laboratory; EMBL, European Molecular Biology Laboratory; gMO, gastric mucin oligosaccharide; HPAEC-PAD, high-performance anion exchange chromatography with pulsed amperometric detection; NCBI, National Center for Biotechnology Information; PGM, porcine gastric mucin.

We next performed individual comparisons between the transcriptomes from gMO, GlcNAc, and GalNAc grown cells to the cellobiose reference to determine if gMO or the two *N*-acetyl sugars elicit specific responses relative to a substrate unrelated to mucin (*i.e*., test the hypothesis that all three previously compared substrates induce genes involved in mucin degradation, leading to few GHs identified in the comparison). The GalNAc–cellobiose comparison revealed 310 genes that were significantly regulated (210 up, 100 down in response to GalNAc). GlcNAc–cellobiose and gMO–cellobiose had fewer genes that were differentially expressed: GlcNAc 156 genes (123 up, 33 down in response to GlcNAc) and gMO 95 genes (52 up, 43 down in response to gMO) with only nine genes in common between them (Figs. 2, *A* and *B*, S2, *E* and *F*, and Tables S5–S7). Overall, only six CAZymes were significantly upregulated across all three substrates relative to the cellobiose reference. There were three GH1 enzymes and a CE4 uniquely expressed during GalNAc growth, whereas two genes encoding putative GH89 and GH13_29 enzymes were the only commonality between all three substrates.

Only 24 genes exhibited expression changes that were unique to growth on gMO, and this list included seven genes in a single upregulated locus that encodes a predicted two-component regulator and a YhfC family protease (UGRO018_01646) (Fig. 2*C* and Fig. S2*G*). Although little is known about the function of this protease family, it is possible that it and surrounding gene products play a role in the degradation of mucin glycoprotein components, although the deduced protease polypeptide sequence does not contain a predicted signal peptide for secretion (67). To test if *Hm* possesses mucin protease activity, gMO-grown cells were incubated with cMUC2 and PGM overnight before analyzing the residual mucin by SDS-PAGE and periodic acid Schiff staining to visualize the mucin glycoprotein. The results of these experiments failed to indicate that *Hm* cells possess the ability to degrade either of the mucin glycoprotein tested (Fig. S2, *H* and *I*).

One of the most highly expressed genes (UGRO018_02407) in all three comparisons (GalNAc, GlcNAc, and gMO relative to cellobiose), and the most highly expressed in GlcNAc and gMO, encodes a putative GH89 family α-*N*-acetylglucosaminidase with four appended CBM32 domains (Fig. 2*B*). The N terminus contains two CBM32 domain homologs, which are divergent from two more well-conserved domains that surround the GH89 catalytic domain (Fig. 2*D*). Expression of this enzyme is notable because mammalian gastric mucins have been shown to be enriched in terminal α-linked GlcNAc compared with mucin from more distal intestinal sites (42, 64, 68, 69) and GH89 family members from other mucin-degrading bacteria (*Clostridium perfringens* and *Bifidobacterium bifidum*) have been reported to cleave GlcNAc(α-1,4)Gal in type III gastric mucin (30, 31). Similar to UGRO018_02407^GH89^, these previously characterized GH89s also contain multiple N- and C-terminal CBM32 domains, which have been shown to bind to components of mucin glycans, whereas the catalytic domains only show 45% and 41% identity, respectively, with UGRO018_02407^GH89^. Recombinantly expressed UGRO018_02407^GH89^ released GlcNAc from both PGM and gMO when measured by high-performance anion exchange chromatography with pulsed amperometric detection (HPAEC-PAD) (Fig. 2*E*) and TLC (Fig. S3*A*). However, no activity on *p*NP substrates was detected, including *p*NP-α-GlcNAc, suggesting that native mucin glycans may be required for activity (Fig. S3*B*). Nonetheless, the observed UGRO018_02407^GH89^ activity on PGM and gMO supports the conclusion that *Hm* uses this mucin-inducible enzyme to target GlcNAc residues in gastric mucins.

A similar gene expression profile was also observed for UGRO018_01830, the predicted member of GH family 13, subfamily 29 (GH13_29) (Fig. 2*B*). The expression of this family/subfamily, which has been shown to have activity on trehalose (Glc(α-1,1)Glc), is puzzling considering gMO does not contain glucose and that GalNAc and GlcNAc also induce expression of this gene compared with cellobiose. Since the comparison of these substrates is to cellobiose (β-1,4-linked glucose disaccharide), it is possible that the metabolism of glucose causes repression of this gene relative to the mucin sugar conditions. However, a quantitative PCR (qPCR) analysis of gene expression during growth in GlcNAc and multiple glucose disaccharides indicated that cellobiose does not elicit repression of UGRO018_01830^GH13_29^, at least with respect to GlcNAc, and trehalose appears to be an activating signal (Fig. S3*C*).

**Figure 3:**
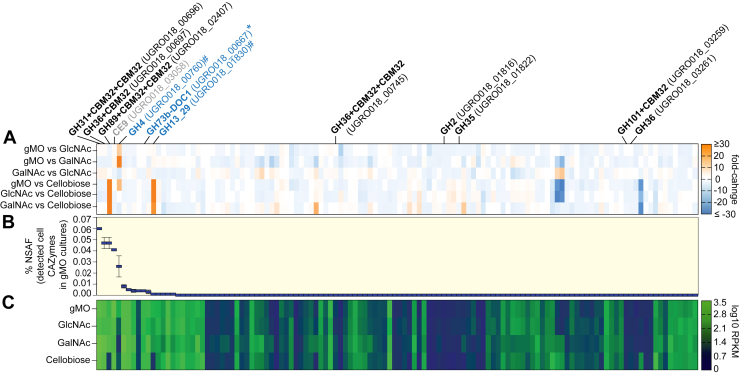
**Proteomics uncovers two constitutively expressed glycoside hydrolases.***A*, heatmap showing fold-change differences of all *Hm* CAZymes from the pairwise RNA-Seq comparisons labeled at the *left* (note that nonsignificant fold changes are also shown for reference, and the order is based on protein abundance results shown in *B*). All GH families known to exhibit mucin glycan– related activities are labeled in *black* on *top* with corresponding locus tag with the exception of three genes that have *blue text*: (1) one gene (GH73b; ∗) with a product implicated in peptidoglycan processing and (2) two other genes (GH13_29, GH4; #) with products not yet associated with mucin glycan utilization. The CE9 (*gray label*) is differentiated from the GHs (*black*). *B*, percent normalized spectral abundance factor (NSAF) based on whole-cell proteomics is shown for all CAZymes in the same order as shown in *A* (n = 2 with values sorted from high (*left*) to low (*right*). *C*, log_10_-transformed RPKM values (from RNA-Seq) for all CAZymes on each growth substrate, also in the same order as *A* and *B*. All locus tags corresponding to a CAZyme in *A*–*C* were sorted from *left* to *right* by %NSAF values in the gMO samples in *B* and are matched vertically among subpanels. CAZyme, carbohydrate active enzyme; GH, glycoside hydrolase; *Hm*, *Hoskinsella mucinilytica*; RPKM, Reads per Kilobase of transcript per Million mapped reads.

Since differential transcription only illuminated one CAZyme (UGRO018_02407^GH89^) that is known to be involved in foraging sugars from mucin glycans (Fig. 3*A*), we decided to investigate whether other functions involved in metabolizing mucin might be constitutively expressed and therefore escape detection by RNA-Seq differential analysis. We performed proteomics on whole cells and cell-free supernatants from cultures grown to midexponential phase on gMO. There were no detectable *Hm* proteins in the cell-free supernatants using either acetone precipitation or a more sensitive pyrogallol red–molybdate–methanol precipitation method (70), indicating that the enzymes used to degrade gMO are likely cell associated and not secreted. Consistent with this, the whole cell proteome measured from acetone-precipitated material detected 908 proteins with three GHs present in the top five overall most abundant proteins by normalized spectral abundance factor (NSAF) (RNA polymerase and pyruvate synthetase were the two others; Fig. 3*B* and Table S8). The differentially expressed GH89 (UGRO018_02407^GH89^), as well as a GH31, subfamily 18 (UGRO018_00696), and a GH36 (UGRO018_00697) represented the most abundant CAZymes identified with NSAFs of 0.047%, 0.060%, and 0.047%, respectively.

**Figure 4:**
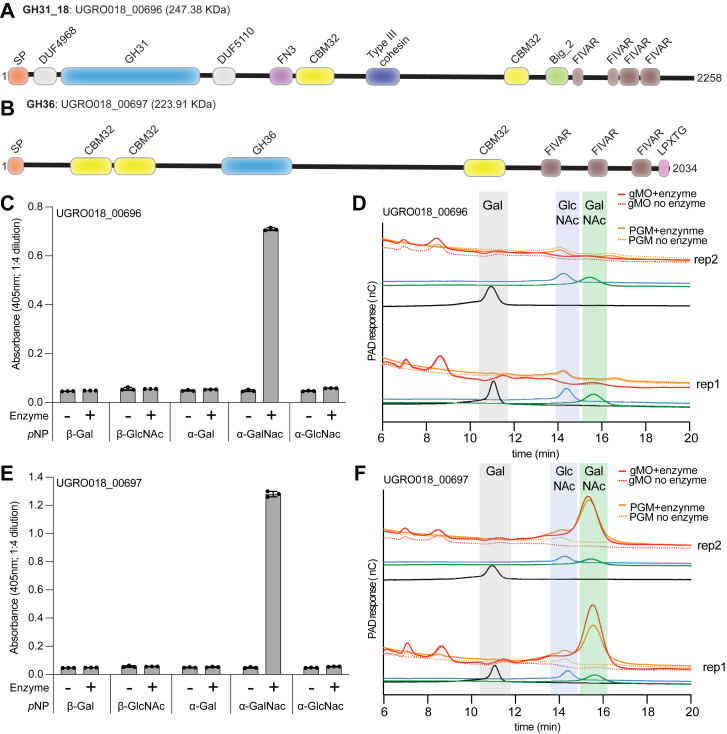
**α-GalNAc-cleaving activities of GH31_18 and GH36 family enzymes.***A*, predicated protein domain structure of the constitutively expressed UGRO018_00696^GH31_18^ from NCBI BLASTp results. *B*, predicated protein domain structure of the constitutively expressed UGRO018_00697^GH36^ from NCBI BLASTp and EMBL-EBI InterProScan results. Domain abbreviations are SP, CBM32, FIVAR, and LPXTG. *C*, overnight endpoint readings (absorbance at 405 nm) of 1:4 diluted samples from *p*-nitrophenyl substrates incubated with recombinantly expressed UGRO018_00696^GH31_18^ enzyme. *D*, HPAEC-PAD chromatograms from overnight incubations of the GH31_18 in gMO or PGM. *Solid lines* indicate the addition of enzyme as compared with the negative controls (*dotted lines*) lacking enzyme. Standards for galactose (*black*), GlcNAc (*blue*), and GalNAc (*green*) were run at 10 μM. *E*, overnight endpoint (absorbance at 405 nm) of 1:4 diluted samples from *p*-nitrophenyl substrates incubated with recombinantly expressed UGRO018_00697^GH36^ enzyme. *F*, HPAEC-PAD chromatograms from overnight incubations on gMO and PGM for the GH36. *Solid lines* indicate the addition of enzyme as compared with the negative controls (*dotted lines*) lacking enzyme. Standards are the same as *D*. Big_2, bacterial Ig-like domain, group 2; CBM32, carbohydrate-binding module 32; DUF, domain of unknown function; EBI, European Molecular Biology Laboratory; EMBL, European Molecular Biology Laboratory; FIVAR, found in various architectures; FN3, fibronectin type III; GH, glycoside hydrolase; gMO, gastric mucin *O*-glycan; HPAEC-PAD, high-performance anion exchange chromatography with pulsed amperometric detection; LPXTG, cell wall anchor domain; NCBI, National Center for Biotechnology Information; PGM, porcine gastric mucin; SP, signal peptide.

To align the transcriptomic and proteomic data, we directly examined the magnitude of the RNA-Seq normalized Reads per Kilobase of transcript per Million mapped reads (RPKM) values instead of measuring fold changes by differential sequence analysis. We sorted all putative CAZyme-coding genes based on protein abundance from high to low (*left* to *right* in Fig. 3*B*) and then displayed log_10_-adjusted RNA-Seq RPKM values (Fig. 3*C*). This analysis revealed that some GHs detected by proteomics, which had little or no differential expression between substrate comparisons, exhibited some of the highest overall expression values. Not surprisingly, the two enzymes that displayed concordantly elevated protein and transcript values were (1) the GH31_18 (UGRO018_00696^GH31_18^; Fig. 4*A*), which contains two CBM32 domains, had the highest RPKM value of any GH yet had a −1.59 fold change when comparing gMO to cellobiose and (2) the GH36 (UGRO018_00697^GH36^; Fig. 4*B*), which also contains one well-conserved CBM32 and two more divergent homologs at the N terminus, had elevated RPKM values but no significant differential expression between gMO and cellobiose (−1.38). There was also a GH73 that displayed minimal gMO/cellobiose fold change (−1.05) but had elevated transcript values and a low detectable protein abundance (0.004%).

The GH31 family includes enzymes active against α-GalNAc, amongst many other activities, from both Gram-negative and Gram-positive human gut bacteria (39, 71). Therefore, GH31 has been recently divided into subfamilies, revealing that the GH31_18 subfamily gathers all members active on α-GalNAc and no other activity so far. The most similar sequences from UGRO018_00696^GH31^ among the GH31 family are indeed all five characterized GH31_18 that are active on α-GalNAc, with 42% to 46% identity (72). Recombinantly expressed UGRO018_00696^GH31_18^ displayed activity against *p*NP-α-GalNAc (Fig. 4*C*) but no activity against more native substrates that contain α-GalNAc, including PGM, gMO, blood group A, Tn Antigen (α-GalNAc linked to serine/threonine), and fetuin (sialylated blood glycoprotein containing *O*-linked glycans with core α-GalNAc linked to serine/threonine) (Fig. S3, *D*–*F*). Further analysis by HPAEC-PAD also failed to reveal activity for UGRO018_00696^GH31_18^ against PGM and gMO substrates in the conditions tested (Fig. 4*D*). Despite the lack of activity on these native mucin substrates, the activity detected on *p*NP-α-GalNAc suggests that this enzyme removes this linkage in some context. Although confirmation of another α-GalNAcase described below also explains how *Hm* removes these linkages.

The GH36 family contains two characterized members with α-*N*-acetylgalactosaminidase activity against mucin glycans, whereas a large majority of GH36 family members display α-Gal activity, some against blood group B (73, 74). Sequence analysis reveals that UGRO018_00697^GH36^ is more similar to the two GH36 enzymes active on α-GalNAc than to any other GH36 targeting α-Gal. To test the specificity of UGRO018_00697^GH36^ for these two linkages, we recombinantly expressed it, determining that it has activity against *p*NP-α-GalNAc but not *p*NP-α-Gal (Fig. 4*E*). This enzyme also released GalNAc from gMO, PGM, blood group A, and Tn Antigen but displayed no activity on an α-Gal-containing blood group B substrate (Fig. S3, *G* and *H*). In addition, HPAEC-PAD confirmed that UGRO018_00697^GH36^ releases GalNAc from both PGM and gMO (Fig. 4*F*). These data indicate that the constitutively expressed UGRO018_00697^GH36^ targets terminal α-GalNAc found in mucin, either as blood group A or peptide-linked Tn antigen.

The GH73 protein (UGRO018_00667) was the fifth most abundant GH and the 10th of all CAZymes. Members of this protein family have been characterized from several bacteria and are present in many species of the human gut. This family includes endo-β1,4-*N*-acetylglucosaminidase enzymes active on either *N*-linked mannosyl glycoproteins or peptidoglycan (75, 76). While we did not measure the growth of *Hm* on *N*-linked glycoprotein substrates, it is possible that this enzyme is involved in peptidoglycan recycling and/or cell division. Although because *Hm* encodes three members of this family, we cannot rule out the possibility that it is involved with scavenging peptidoglycan sugars from other organisms' cell walls, which would also align with the strong growth on *N*-acetylmuramic acid (Fig. 1).

Interestingly, the GH4 (UGRO018_00760^GH4^) that displayed increased transcription during growth on gMO was also present with an NSAF of 0.026%, making it the fourth most abundant protein for both GHs and all CAZymes. Alternatively, the GH13_29 that was predominantly responsive to growth on trehalose was detected at only 0.001%. Other CAZymes detected included a CE family 9 (UGRO018_03058; 0.04%) and a glycosyl transferase family 5 (UGRO018_00925; 0.008%). Members of these two families have been characterized as GlcNAc-6-phosphate deacetylase and α-glucan synthase, respectively (77, 78) but we did not perform recombinant enzyme experiments to measure their activities.

Even though *Hm* does not appear to encode fucosidases or sialidases in known families, it does encode the two predicted sulfatases noted above. Both sulfatases were not significantly differentially transcribed in the RNA-Seq comparisons, nor were they detected by proteomics, suggesting that they are not critical during the growth conditions we tested. Taken together, our multiomics analysis reveals that in the presence of gMO, *Hm* differentially expresses a gene encoding a GH89 enzyme with activity toward α- GlcNAc and a GH4 protein with an unknown substrate (the former in response to gMO, GlcNAc, and GalNAc and the latter mostly in response to gMO) with the constitutively expressed GH31_18 and GH36 enzymes functioning to target α-GalNAc, although the native substrate target for the GH31_18 is undetermined.

### *O*-glycan analysis confirms *Hm* selectively utilizes *N*-acetylhexosamines from gMO

**Figure 5:**
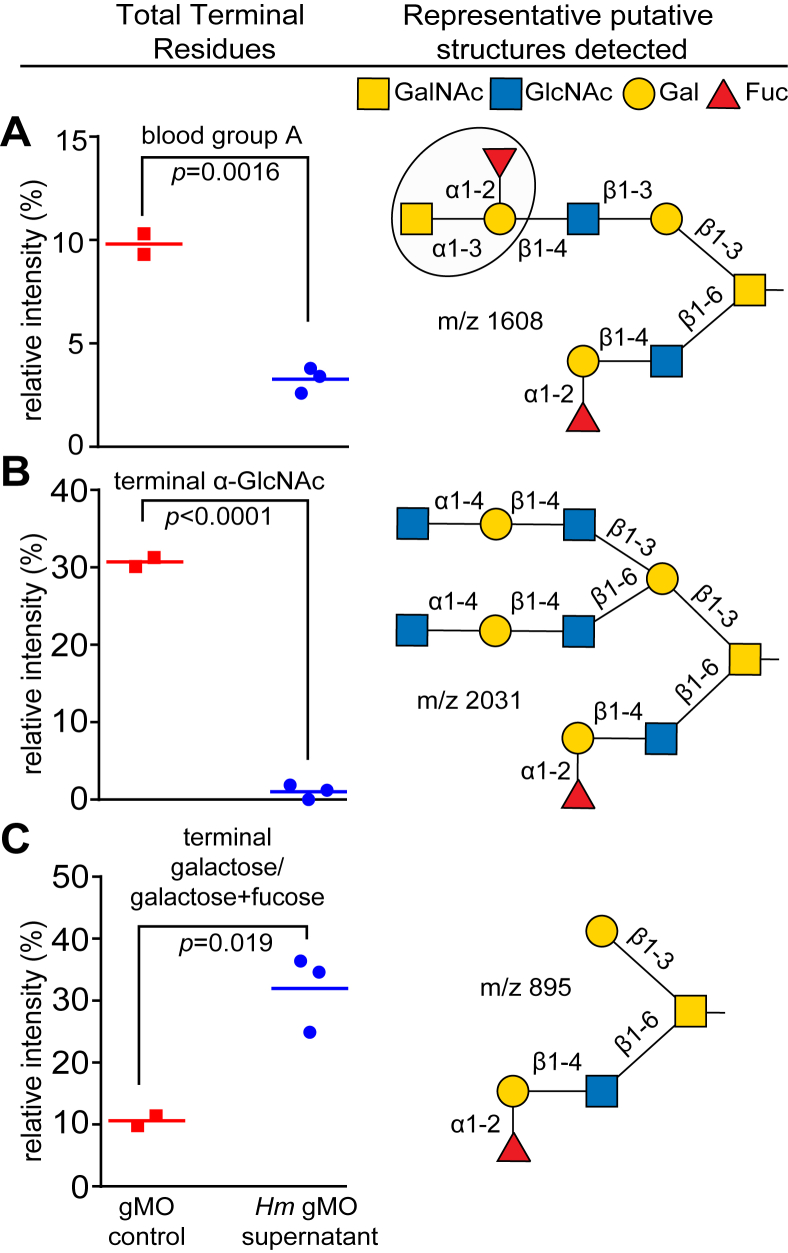
**Mass spectrometry reveals selectivity for *N*-acetylhexosamine degradation by *Hm*.** The relative abundance of three groups of gMO *O*-glycans was measured before and after *Hm* growth using LC–MS/MS. *A*, blood group A (*circled trisaccharide* in representative structure). *B*, terminal α-linked GlcNAc. *C*, galactose/galactose + fucose residues. Structures at the *right* show only representative structures with these terminal sugars/linkages. The *m/z* ratios shown are only for the representative putative structures shown in *A*–*C*. See Table S9 for a full list of deduced gMO glycans detected. The *p* values shown in plots at the *left* are based on a paired two-tailed Student’s *t* test). gMO, gastric mucin *O*-glycan; *Hm*, *Hoskinsella mucinilytica.*

Based on its specificity for growth on gastric mucin and associated *O*-glycans, we next characterized how *Hm* degrades gMO from PGM. Since *Hm* is unable to grow on Fuc or NeuNAc, and known α-fucosidase and sialidase families appear to be absent in the genome, we hypothesized that residual gMO after *Hm* growth would contain increased proportions of oligosaccharides with terminal Fuc and/or NeuNAc and diminished abundance of glycans with terminal GalNAc and GlcNAc. Preliminary TLC on cell-free supernatants of gMO grown *Hm* cultures, relative to uninoculated controls, suggested both the disappearance of higher molecular weight *O*-glycans and the appearance of new structures that were difficult to distinguish as oligosaccharides or NeuNAc, suggesting partial degradation by *Hm* (Fig. S4*A*). However, there did not appear to be abundant presence of any of the four neutral sugars, including Fuc and Gal. Given this preliminary result, we next performed LC–MS/MS to provide better resolution of the remaining oligosaccharides present in supernatant. In total, 67 *O*-glycan species were resolved across all samples, with fewer in *Hm* gMO supernatants than gMO media controls, indicating that some glycans had been degraded (Table S9). The *O*-glycans detected in the undigested gMOs primarily contained terminal β-1,4-Gal, α-1,2-Fuc, α-1,4-GlcNAc, and α-1,3-GalNAc (blood group A). Glycans with terminal NeuNAc were not detected, reflecting our purification method that involves anionic exchange chromatography to remove charged glycans (16). After growth of *Hm* on the gMO mixture, the level of blood group A oligosaccharides (terminal α-1,3-GalNAc) was only 3% compared with 10% in the undigested controls (*p* = 0.0016) (Fig. 5*A*). More strikingly, the level of terminal α-1,4-GlcNAc was reduced from 31% to 1% (*p* < 0.0001) after *Hm* growth (Fig. 5*B*). In contrast, *O*-glycans terminating with Gal, Gal, and Fuc together increased from 11% to 32% (*p* = 0.019) (Fig. 5*C*). In addition, the amount of terminal Fuc remained relatively unchanged between *Hm* supernatants and media controls at 84% and 85%, respectively. These data are concordant with the ability of *Hm* to release terminal GalNAc and GlcNAc, leading to an increase in β-linked Gal and unscathed terminal Fuc residues.

An increase in terminal β-linked *N*-acetylhexosamines was not detected by mass spectrometry (MS) after *Hm* growth, and the abundance of glycans presenting terminal Gal increased, suggesting that *Hm* is poorly equipped to remove β-linked Gal. This is consistent with the observation that *Hm* only grows weakly on LacNAc (Gal β-1,4 GlcNAc) (Fig. 1*E*). To extend this observation, we also tested tetra LacNAc [Gal β-1,4 GlcNAc β-1,3-]_4_ to determine if either β-linkage between these sugars could be hydrolyzed by *Hm* in an endo- or exocatalytic fashion. Growth profiles for LacNAc again showed a small initial growth phase followed by a slow increase in absorbance, whereas no growth on tetra LacNAc was observed (Fig. S4*B*). A subsequent TLC on supernatants showed that the LacNAc media–only control contained small amounts of contaminating monomeric GlcNAc, and the *Hm* supernatant appeared to contain a “precipitate.” Thus, we conclude that the residual GlcNAc likely drives the initial growth, and the precipitate results in a slow increase in absorbance over time (Fig. S4*C*). Overall, these data further support our conclusion that *Hm* is adept at targeting terminal α-linked GalNAc and GlcNAc residues from gMO but not Fuc or β-linked Gal.

### Metabolite analysis reveals acetate and butyrate as the major *Hm* fermentation products

**Figure 6:**
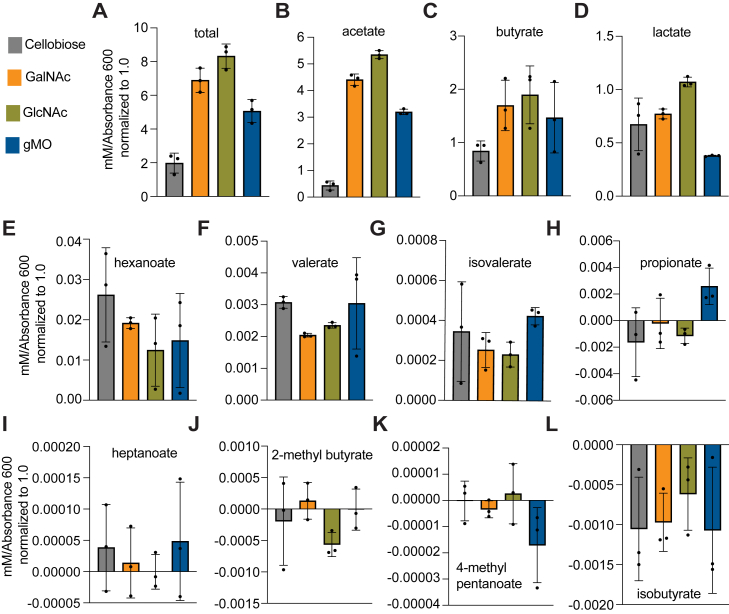
**Metabolite analysis shows butyrate production from *Hm* fermentation.** SCFA analysis for cultures grown using cellobiose, GalNAc, GlcNAc, or gMO as the sole carbon source and represented in descending total concentrations detected as *A-L*. SCFA concentrations in culture supernatants were normalized to an optical density of 1.0 at an absorbance of 600 nm. Error bars represent the SDM (n = 3). gMO, gastric mucin *O*-glycan; *Hm*, *Hoskinsella mucinilytica*; SCFA, short-chain fatty acid.

Anaerobic gut bacteria produce short-chain fatty acids during carbohydrate fermentation that are absorbed in the large intestine and can have beneficial impacts directly in the colon and throughout the body. Thus, we set out to determine *Hm*’s fermentation profile after growth on cellobiose, GalNAc, GlcNAc, and gMO. After normalizing to media-only controls and an absorbance of 1.0 at 600 nm, the most abundant metabolites detected were acetate, butyrate, and lactate (Fig. 6, *A*–*D* and Table S10). Other fatty acids, including hexanoate, valerate, isovalerate, propionate, heptanoate, 2-methyl butyrate, 4-methyl pentanoate, and isobutyrate, were only observed in trace amounts or not detected regardless of growth substrate (Fig. 6, *E*–*L*). Although many bacteria in the intestine produce acetate, butyrate production is more restricted, with only an estimated 20% to 30% of human gut bacteria, mostly from the Firmicutes phylum, containing known pathways to produce butyrate (79). Butyrate production from the fermentation of mucin or mucin-like glycans, like human milk oligosaccharides, is also uncommon (80). Here, we present a newly identified Gram-positive micro-organism capable of contributing to the overall butyrate pool *via* mucin glycan metabolism.

### *Hm* prevalence is low in human stool surveys

*Hm* was only recently isolated (60). To determine *Hm* presence within and across individuals, we searched multiple databases. A National Center for Biotechnology Information (NCBI) nucleotide BLAST at the time of isolation revealed that the only matching hit with >97% near full-length 16S rRNA gene identity was in a culture-independent mucosal biopsy clone library from a patient with IBD (NCBI GenBank accession ID: EF071180.1). Close relatives of *Hm* also seem to be uncommon in sequence-based human stool microbiota surveys. In a search against the 16S rRNA V4 region of 1817 IBD stool samples, only 0.17% (3/1817) of samples contained matching sequences (81). Further searches against samples from the Personalised Responses to Dietary Composition Trial (PREDICT 1) (82) and an introductory biology laboratory course at the University of Michigan (55, 83) also revealed that *Hm* is low in both prevalence and abundance when present, in these samples (Table 1). As a control comparison, we used the same method to search for exact matches for two well-studied mucin-degrading bacteria, *B. thetaiotaomicron* and *A. muciniphila*, revealing that they are both higher in abundance and prevalence: *B. thetaiotaomicron* was on average 248-fold more prevalent and 463-fold more abundant in these two databases; *A. muciniphila* was on average 102-fold more prevalent and 569-fold more abundant in these two databases. Although the abundance of *Hm* within feces-based microbiota composition surveys is apparently low, there may be mucin-associated niches that are difficult to sample or that are not well represented in stool-based surveys. Further work is required to illuminate the contributions of potentially low-abundance microbes, such as *Hm*, especially those involved in host mucin degradation, and how their presence affects other members of the microbiota.

**Table 1:** Prevalence and abundance of 16s rRNA gene V4 region Amplicon Sequence Variant sequences of three mucin-degrading bacteria in human fecal samples

Bacterial species	Database	Samples present	Total samples	% Positive	% Abundance (when positive)
*Akkermansia muciniphila*	Bio 173	1954	6292	31.06	0.502
*Akkermansia muciniphila*	Predict	743	1098	67.67	0.461
*Bacteroides thetaiotaomicron*	Bio 173	5269	6292	83.74	0.670
*Bacteroides thetaiotaomicron*	Predict	1017	1098	92.62	0.315
*Hoskinsella mucinilytica*	Bio 173	12	6292	0.19	0.013
*Hoskinsella mucinilytica*	Predict	18	1098	1.64	0.00036

## Discussion

One element that contributes profoundly toward shaping the composition of each individual’s microbiota is nutrient acquisition from the host and dietary carbohydrates. While diet is a dynamic feature, the consistently secreted host mucin glycoproteins along the GI tract create a more continuously present carbon source. Several studies have investigated bacterial regulatory and enzymatic strategies to utilize mucin, and it is becoming apparent that individual bacteria have evolved to access components of this complex glycoprotein differently. Some species tightly regulate expression of the enzymes involved, whereas others constitutively produce them, and some species secrete the enzymes involved, whereas others retain them on the cell surface or in the periplasm (16, 17, 25, 38, 84). Some species discard the carbon-rich sugar NeuNAc, whereas others modify it during cleavage from the *O*-glycan terminus so it becomes unavailable to other species (29, 65, 85, 86). Our culture-based approach identified a novel butyrate-producing Gram-positive bacterium, *Hm*, capable of growth on both intact gastric mucin and its associated glycans. Interestingly, this isolate is only capable of exploiting the α-linked *N*-acetylhexosamine sugars, GalNAc and GlcNAc, and concordantly employs a limited assemblage of mucin-degrading CAZymes. Since *Hm* is adept at removing these sugars, even on intact PGM, the remaining pruned gastric mucin oligosaccharides may alter the way other bacteria interact with these modified mucins either as nutrient sources or physical interactions (*e.g*., binding to the modified terminal sugars).

Together, our results add to a growing ensemble of studies into how mucins can be foraged by bacteria in the human GI tract and the genes involved. The *Bacteroides* species employ dozens of discrete genetic loci called polysaccharide utilization loci to target gMOs, and *A. muciniphila* contains mucin utilization loci. Both species’ gene clusters encode protein complexes to transport products *via* TonB-dependent transporters (25, 33). Although *B. thetaiotaomicron* has evolved to use gMOs, most or all the >10 nonmucin polysaccharides it can use are preferred energy sources over *O*-glycans (87) and it is poorly equipped to use intact mucin glycoprotein (16). Other *Bacteroides*, including *Bacteroides fragilis* and *Bacteroides massiliensis*, which have restricted fiber metabolism profiles compared with *B. thetaiotaomicron*, can have variable and even reciprocal preferences for using gMOs (26). This leads to the idea that some species, notably *Akkermansia mucinphila*, have evolved as glycan specialists and have become highly efficient at degrading mucin glycoprotein, even to the core GalNAc attached to the mucin polypeptide (17). At least two *Ruminococcus* species (*R. torques* and *R. gnavus*) are also capable of degrading intact MUC2 (16, 46). *R. gnavus* encodes a sialidase that modifies NeuNAc during cleavage so that it is unavailable to other species and imports the modified sugar *via* ABC transporters (86). The closely related *R. torques* constitutively secretes a repertoire of ∼20 mucin-degrading enzymes into its surrounding environment (16, 29). Studies on mucin-degrading *Bifidobacterium* species have shown that they can utilize a variety of adhesins to target mucins in addition to CBM domains (88). While these examples are not an exhaustive list, it is important to note that each bacterial species exhibits some amount of novelty in its substrate range, gene regulation, or catalytic approach (including the breadth of mucin-degrading enzymes it possesses). In this study, we provide insight into how *Hm*, a mucin specialist in the human gut microbiota, may persist by only deploying a small set of mucin-degrading enzymes that allow it to harvest exposed *N*-acetylhexosamines from a particular source of mucins (gastric and not colonic).

Pioneering studies on the gut microbiota by Cromwell and Hoskins (89), Hoskins and Boulding (90), and Salyers *et al.* (91, 92) laid the foundation for studies that untangle the molecular connections between diet- and host-derived nutrients and gut bacteria that use them, sometimes in a host genotype–specific fashion. The nutrient-niche hypothesis formulated by Rolf Freter *et al.* proposed “that each of the several hundred bacterial species which comprise the intestinal ecosystem is controlled by one or a few nutritional substrates which this strain can utilize most efficiently in the presence of H_2_S and at the conditions of pH and anaerobiosis prevailing in the intestine” (93, 94). Although these concepts continue to be built upon today (23, 95) and include other factors, such as secondary metabolites (96, 97), a fundamental element is that the abundance of each bacterial species’ key nutrient may set the carrying capacity for that species. Thus, the low abundance of *Hm* in the datasets analyzed may be attributable to its preferential use of gastric mucins, which might either lead to its niche being located in more proximal areas of the GI tract in which the microbiota exists (*e.g*., ileum instead of distal colon) that receive shed gastric mucins or by smaller amounts of these mucins entering its niche. Moreover, the genotype of fecal donors may be a limiting factor in determining a species presence, especially for mucin glycan specialists only capable of targeting terminal monosaccharide residues presented as a specific blood group. Any of these features or a combination of them could reduce detection in stool-based surveys because *Hm* numbers would be reduced in its native niche and possibly diluted during colonic transit. Ultimately, the restricted use of GlcNAc and GalNAc from only gastric mucins may limit the ability of *Hm* to expand to other niches, along the intestines or to new hosts.

As deeper collections of gut bacterial isolates are created and novel species are cultivated, we gain a better understanding of not only the diversity of microorganisms that reside within the human gut but also the determinants of how they flourish. Defining whether new organisms, such as *Hm*, play a role in affecting how the niches are shaped within the mammalian gut requires further investigation. It will be fundamental for future studies to examine whether specific bacterial strains are capable of creating microbiota-modified carbohydrates that may affect carbon sequestration or the regulatory signals (*i.e*., modified *O*-glycans) of other gut bacteria.

## Experimental procedures

### Bacterial growth and high-throughput phenotypic assays

All growths were performed in a Coy anaerobic chamber at 37 °C with an atmosphere of 85% N_2_, 10% H_2_, and 5% CO_2_. *Hm* was isolated from a healthy human fecal sample by enriching in a custom chopped meat broth prior to streaking 10 μl onto a depleted media agar plate containing 1% PGM to isolate colonies (98). An individual colony was thrice streaked onto brain heart infusion agar plates supplemented with 10% defibrinated horse blood (Quad Five) to ensure purity prior to growing a single colony in chopped meat broth. However, further testing determined that Gifu anaerobic agar (HIMEDIA) plates provided the best rich media growth. Phenotypic growth measurements were performed as previously described using a BioStack 2WR Plate Stacker coupled to a Powerwave HT spectrophotometer (BioTek) with 200 μl cultures in 96-well clear flat-bottom plates (Costar) in a depleted medium with most substrates sourced from Sigma and Megazyme (56, 62). The custom mucin substrates, PGM, gMO, cMO, and cMUC2, were prepared as previously described (16) whereas bovine submaxillary mucin (Sigma), *N*-acetylmuramic acid (Sigma), and LacNAc (Carbosynth) were sourced from respective suppliers. The mu maximum growth rates of *Hm* on different substrates were measured using the “Estimation of Growth Rates with Package growth rates” as described by Thomas Petzoldt (https://tpetzoldt.github.io/growthrates/doc/Introduction.html). See Table S1 for all substrates tested, total growth yields, and rates.

### RNA extraction and RNA-Seq

For RNA-Seq experiments, cells were grown to midlog phase on respective substrates and centrifuged to pellet bacteria. Cell pellets were resuspended in 1 ml RNA Protect Bacteria Reagent (Qiagen) and transferred to a 2 ml screw cap tube. After incubating for 5 min at room temperature, cells were centrifuged at 7000*g* for 10 min at room temperature, supernatants were decanted, and stored at −80 °C. Cell pellets were thawed on ice, ∼200 μl worth acid washed glass beads (Sigma), 500 μl buffer A (200 mM NaCl, 200 mM Tris, 20 mM EDTA, pH 8.0), 210 μl 20% SDS, and 500 μl phenol:chloroform:isoamyl alcohol (IAA) (125:24:1; pH 4.5) were added to each tube and bead beated for 3 min. Tubes were centrifuged for 3 min at 18,000*g* at 4 °C, and the aqueous phase was removed into a new RNase-free tube. Another 500 μl phenol:chloroform:IAA was added, mixed, and centrifuged as above. The aqueous phase was removed, 0.1 volume of 3 M sodium acetate (pH 5.5) and 600 μl cold 100% isopropanol were added and mixed by inversion. After precipitating at −80 °C for 20 min, RNA was pelleted by centrifuging at 18,000*g* for 20 min at 4 °C. Supernatants were discarded, and RNA pellets were washed with 70% isopropanol prior to centrifuging at 18,000*g* for 5 min at 4 °C. Supernatants were discarded, and pellets were dried at room temperature before resuspending in 86 μl nuclease-free water. RNA was subjected to DNaseI treatment (New England Biolabs) with 4 μl prior to reprecipitating and pelleting of RNA as above. Finally, RNA pellets were washed with 1.3 ml 70% isopropanol, air-dried, and resuspended in 50 μl RNase-free water. Total RNA was quantified using Qubit 2.0 (Invitrogen).

To ensure a DNA-free RNA extraction that would be successful for RNA-Seq, qPCR was performed on all RNA samples using a standard curve of genomic DNA and 16S rDNA gene-specific primers. All samples contained <0.5 ng/μl of genomic DNA, were subjected to rRNA depletion, and residual mRNA was quality controlled and converted to sequencing libraries at either the University of Michigan Advanced Genomics Core using an Illumina Novaseq 6000 or SeqCenter using an Illumina Novaseq X. Barcoded data were demultiplexed and analyzed using Arraystar software (DNASTAR, Inc) using RPKM normalization with default parameters. Gene expression during growth on each substrate was compared against all other substrates. Genes that were significantly upregulated or downregulated were determined as those with an average fold change >5-fold and a *p* value <0.05 (Student’s *t* test with Benjamini–Hochberg correction for false discovery).

### Recombinant enzyme expression and activity assays

A 1 ml aliquot of overnight-grown *Hm* cells was pelleted, and DNA was extracted using the DNEasy Blood and Tissue kit (Qiagen) with the optional Gram-positive step. Purified DNA was quantified on a Nanodrop (Thermo) prior to PCR. DNA primers were designed to produce an amplicon spanning the start codon for the GH4 (no signal peptide) and the next codon after the predicted signal peptide cleavage site for the GH31_18, GH36, and GH89 to the next-to-last codon (a stop codon is included in the vector below). PCR was performed using Phusion High-Fidelity DNA Polymerase (Thermo) according to the manufacturer’s protocol.

A gel-extracted, column-purified PCR product for each respective enzyme was ligated into the pETite vector containing a C-terminal His_6_ tag (Lucigen) and transformed into HI-Control 10G cells. After a 1 h recovery in LB broth at 37 °C, cells were plated onto LB plates with kanamycin (30 μg/ml) overnight at 37 °C. The DNA sequence fidelity was confirmed prior to transformation into TUNER *Escherichia coli* cells and plated onto LB with kanamycin. A single colony was picked into LB broth with kanamycin and incubated at 37 °C overnight. A 1 l flask of Terrific broth was inoculated with 10 ml of stationary phase culture and incubated at 37 °C with shaking at 200 rpm until midlog (absorbance of 0.6 at 600 nm) when it was induced with 0.2 mM final IPTG. Enzymes were expressed with 200 rpm shaking at 16 °C overnight, then pelleted, and stored at −80 °C until purification.

Enzymes were purified by centrifuging sonicated cells in Talon buffer (20 mM Tris, 300 mM NaCl, pH 8.0) at 8000*g* and purified on a HisPur Cobalt Resin (Thermo Scientific). Two separate elutions (4 ml [E1] and 4 ml [E2]) were performed and run on an SDS-PAGE gel to verify expression/purification. Soluble, expressed proteins were dialyzed overnight using a 3.5 KDa membrane in Talon buffer.

The purified mucin oligo substrates used in the enzyme or growth assays were sourced from Elicityl (blood group A, blood group B, and tetra LacNAc), extra (Tn antigen and Gal α-1,4 Gal β-1,4 GlcNAc), Carbosynth (LacNAc and Lacto-N-biose), and Sigma (fetuin). The GH4 activity assays were performed in 50 mM Tris buffer (pH 8.0) containing variable amounts of NAD, MnCl_2_, and DTT, as GH4 enzymes have been shown to require these cofactors, with 1 μM of enzyme and incubated overnight at 37°C. The GH family 31, 36, and 89 gMO and PGM reactions (200 μl) contained final concentrations of 1 μM enzyme, 1% substrate, and 50 mM Tris (pH 8.0), and were incubated overnight at 37 °C. Oligosaccharide (blood group A, B, fetuin, and Tn antigen) activity screens for the GH31_18 and 36 were performed in 10 μl reactions with the following final concentrations: 2 μM enzyme, 2 mM substrate, and 50 mM Na_2_PO_4_ buffer (pH 7.0) with overnight incubation at 37 °C. Assays were heat inactivated and subjected to TLC to check for enzymatic activity as previously reported (16). All *p*NP assays were performed in 100 μl reactions with final concentrations of 1 μM enzyme, 1 mM of individual *p*NP substrate, and 100 mM Tris (pH 8.0). Endpoint (24 h) readings of 1:4 diluted samples (to obtain in values under saturation) were obtained in optically clear, flat-bottom, 96-well plates (Costar) at 405 nm using a Synergy plate reader (Biotek).

HPAEC-PAD (Dionex ICS-6000 HPIC System; Thermo Scientific) was performed on 1:10 diluted samples in water using a CarboPac PA100 column (Thermo Scientific) as previously reported (16). Buffers included buffer A (100 mM NaOH), buffer B (100 mM NaOH and 500 mM sodium acetate), and buffer C (water). Data were analyzed in Chromeleon Chromatography Data System Software (Thermo Fisher) and exported for visualization in GraphPad Prism (GraphPad).

### Periodic acid Schiff–stained gels

Cultures were mixed (3:1) with Laemmli buffer and incubated at 98 °C for 10 min prior to electrophoresis on a 4% to 12% gradient PAGE Gel (Invitrogen NuPAGE) for 60 min at 180 V. The following was performed with shaking: The gel was fixed in 50% methanol for 30 min and rinsed three times in 5% acetic acid for 10 min each. Next, the gel was added to 0.7% periodic acid in 5% acetic acid for 3 h and then rinsed three times in 5% acetic acid for 5 min each. Finally, the gel was incubated in Schiff stain (Sigma) overnight and imaged the next day.

### qPCR on *Hm* GH13_29 gene

*Hm* was grown in triplicate on GlcNAc as a sole carbon source (absorbance values of ∼0.6 at 600 nm). Bacterial samples were washed in no-carbon media and transferred to minimal media containing respective carbon sources for 4 h. RNA was preserved with RNAProtect (Qiagen), and total cellular RNA was extracted using phenol:chloroform:IAA (125:24:1; pH 4.5), including DNAse treatment (New England Biolabs). Complementary DNA was synthesized using the SuperScript III Reverse Transcriptase kit (Invitrogen). Transcript measurement of UGRO018_01830^GH13_29^ was performed on a Bio-Rad CFX Connect Real-Time System using a homemade SYBR Green–based master mix (99). Transcript abundance in each sample was calculated using the ddCT method with 16S rRNA transcript normalization.

### Proteomics

After growing *Hm* in 25 ml of depleted medium -gMO (n = 2) to midlog, cells were centrifuged, washed in PBS, and frozen at −80 °C. Supernatants were filtered through a 0.22 μm filter and also stored at −80 °C. Both the cell pellet and supernatants were lyophilized prior to protein extraction.

Cell pellets were resuspended in 500 μl lysis buffer (4% SDS, 50 mM Tris, 10 mM DTT, pH 8.5), heated to 95 °C for 10 min, cooled to room temperature, and centrifuged for 2 min at 4000*g* to remove cell debris/unlysed cells. Ice-cold acetone was added at four volumes to one volume of retained supernatant and precipitated overnight at −20 °C. Proteins were centrifuged for 10 min at 4000*g*, and the resulting pellets were washed in 80% acetone prior to centrifuging at 17,000*g* for 10 min at 4 °C. Supernatants were removed, and the protein pellet allowed to air dry prior to resuspension in 75 μl of proteomics buffer (8 M urea, 50 mM Hepes, pH 8). Approximately 10 ml of freeze-dried supernatant solids were resuspended in 800 μl of water and added to 800 μl of PRMM solution (20% methanol, 0.05 mM pyrogallol red, 0.16 mM sodium molybdate, 1 mM sodium oxalate, 50 mM succinic acid, pH 2) and adjusted to ∼pH 3 using HCl and pH test strips. Resulting solutions were incubated at room temperature for 2 h and then 4 °C overnight. Tubes were centrifuged at 10,000*g* for 60 min at 4°C and washed in 500 μl ice-cold acetone. The centrifugation and acetone wash were repeated, and air-dried pellets were resuspended in 30 μl proteomics buffer (8 M urea, 50 mM Hepes, pH 8).

Cell and supernatant protein dilutions were run on a 4% to 12% Bis–Tris NuPAGE gel (Invitrogen) and subjected to silver staining (Invitrogen). Proteins were then submitted for Shotgun Proteomics (protein ID + 90 min LC–MS/MS analysis) at the University of Michigan Proteomics Resource Facility.

### Analysis of gastric mucin glycoprotein released gMOs using LC–electrospray ionization/MS

Released glycans were resuspended in water and analyzed by LC–electrospray ionization (ESI) tandem MS. The oligosaccharides were separated on a column (10 cm × 250 μm) packed in-house with 5 μm porous graphite particles (Hypercarb, Thermo-Hypersil). The oligosaccharides were injected onto the column and eluted with an acetonitrile gradient (buffer A, 10 mM ammonium bicarbonate; buffer B, 10 mM ammonium bicarbonate in 80% acetonitrile). The gradient (0–45% buffer B) was eluted for 46 min, followed by a wash step with 100% buffer B, and equilibrated with buffer A in next 24 min. A 40 cm × 50 μm i.d. fused silica capillary was used as a transfer line to the ion source. The samples were analyzed in negative ion mode on a LTQ linear ion trap mass spectrometer (Thermo Electron), with an IonMax standard ESI source equipped with a stainless steel needle kept at −3.5 kV. Compressed air was used as a nebulizer gas. The heated capillary was kept at 270 °C, and the capillary voltage was −50 kV. Full scan (*m/z* 380–2000, two microscans, maximum 100 ms, target value of 30,000) was performed, followed by data-dependent MS^2^ scans (two microscans, maximum 100 ms, target value of 10,000) with normalized collision energy of 35%, isolation window of 2.5 units, activation *q* = 0.25 and activation time = 30 ms). The threshold for MS^2^ was set to 300 counts. Data acquisition and processing were conducted with Xcalibur software (version 2.0.7; Thermo Scientific). Glycan structures were visualized in Figure 3 using GlycoGlyph (https://glycotoolkit.com/Tools/GlycoGlyph/).

### Volatile fatty acid measurements

A 1 ml aliquot of gMO, GalNAc, GlcNAc, and cellobiose grown cultures was centrifuged at 10,000*g* for 1 min to pellet cells and passed through a 0.22 μm filter to remove any remaining debris. Supernatants were quantified for fatty acid content using a previously described method (100) at the Michigan Regional Comprehensive Metabolomics Resource Core.

## Data availability

Genome sequence read data can be accessed through the National Institutes of Health NCBI database, NCBI Bioproject: PRJNA1125895 (16S rRNA, PP944848). All RNA-Seq data can be accessed at the NCBI Gene Expression Omnibus database under GSE267699 with samples GSM8273269–83. The MS proteomics data have been deposited to the ProteomeXchange Consortium *via* the PRIDE (101) partner repository with the dataset identifier PXD056157. LC–ESI/MS data from gMO before and after growth by *Hm* are deposited in GlycoPOST https://glycopost.glycosmos.org/preview/10224120786810b294cb9fe (PIN: 5670). All gene expression, proteomic, and MS data graphed in the figures is provided in the associated supporting tables.

## Supporting information

This article contains supporting information.

## Conflict of interest

N. A. P. is a research specialist in the Martens Laboratory at the University of Michigan. The research focuses on understanding the mechanisms that members of the human gut microbiota employ to depolymerize complex carbohydrates and the regulatory circuits involved in nutrient acquisition strategies. The love of science of N.A.P. does not fade away; thanks in part to the Grateful Dead and discovering the wonders of nature. All other authors declare that they have no conflicts of interest with the contents of this article.
