# Supplementary material for: The butyrate-producing Gram-positive human gut bacterium, Hoskinsella mucinilytica, selectively targets host mucin N-acetylhexosamines[image]
Source: J Biol Chem. 2026 Mar 17;302(5):111371. doi: 10.1016/j.jbc.2026.111371 (PMC13096917; doi:10.1016/j.jbc.2026.111371)
Supplement: Supporting Information [file mmc1.pdf]

Supplemental Figures

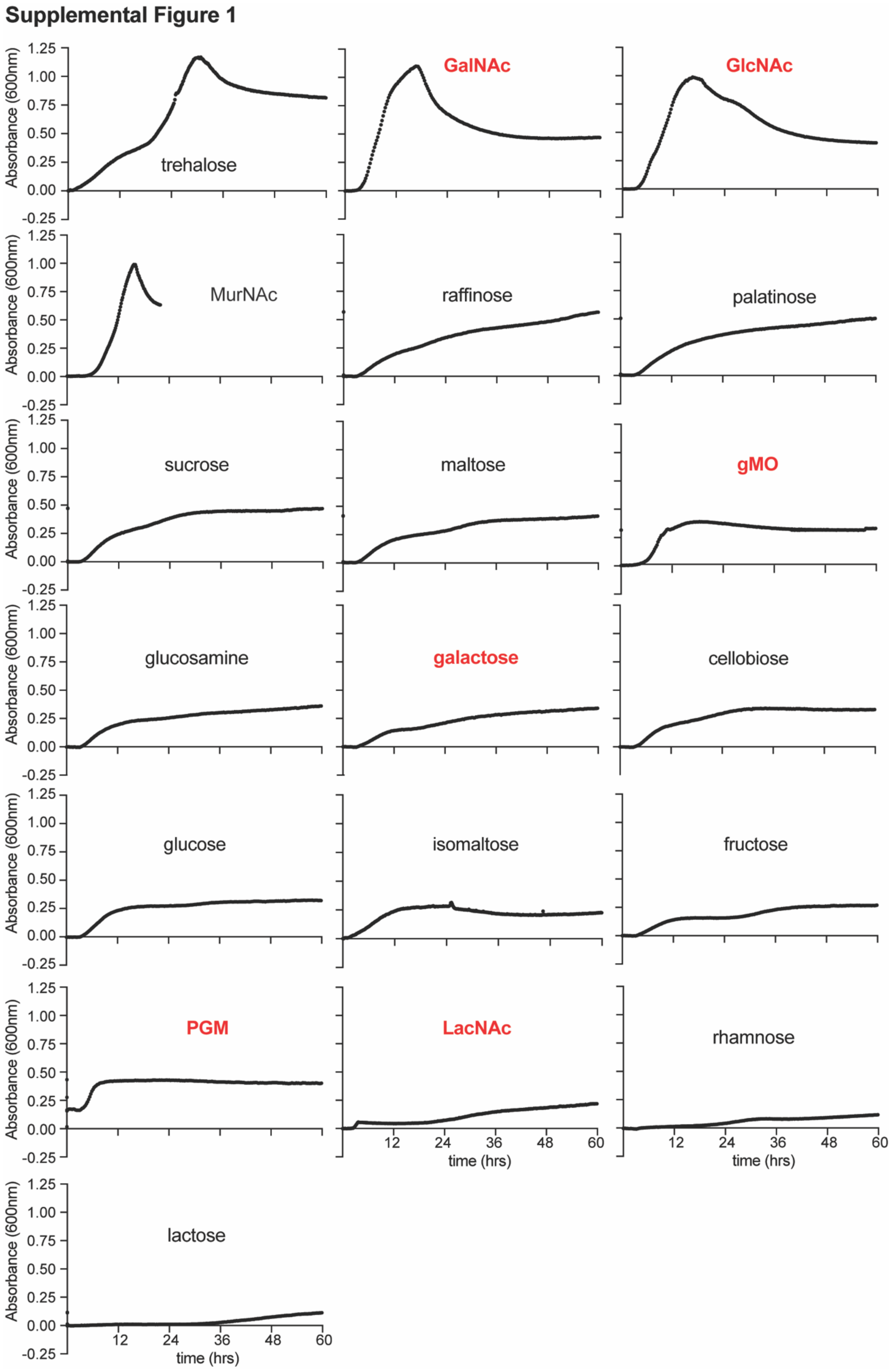

Supplemental Figure 1. Growth profiles over time for carbon sources that *Hm* is capable of growing on to an absorbance (600nm) >0.1 when compared to a water control n=6 except LacNAc (n=3)**.** Red text indicates mucin glycans or mono- or di-saccharides found in mucins.

Supplemental Figure 2.

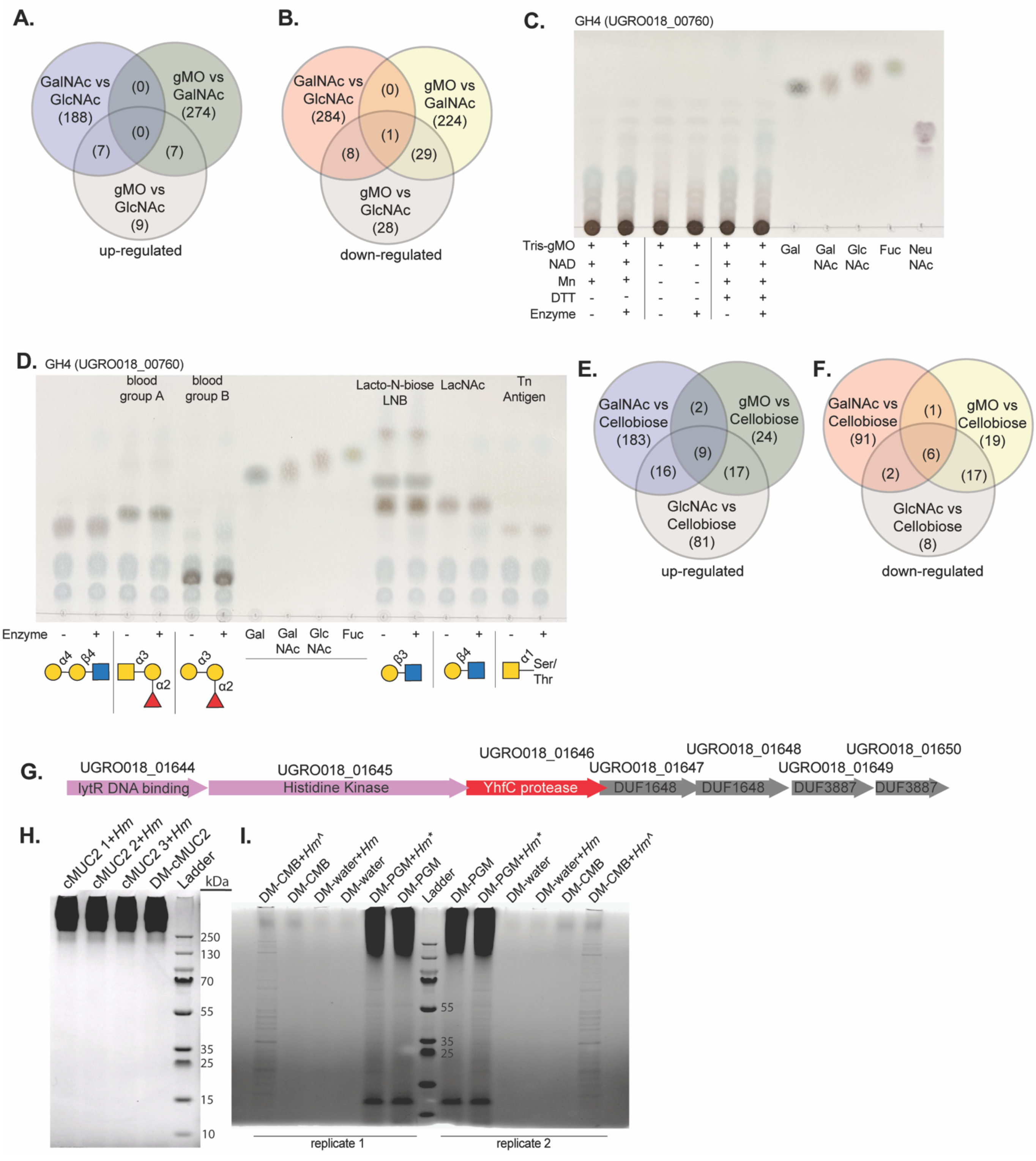

Supplemental Figure 2. (A.) A venn diagram showing significantly up-regulated genes when grown in GalNAc, GlcNAc and gMO. Significance thresholds include having a fold-change >5-fold with a p-value <0.05. **(B.)** A venn diagram showing significantly down-regulated genes when grown in GalNAc, GlcNAc and gMO. Significance thresholds include having a fold-change <5-fold with a p-value <0.05. **(C.)** TLC of the recombinantly expressed gMO-responsive UGRO018_00760^{GH4} enzyme after incubation overnight with gMO. **(D.)** TLC of the recombinantly expressed gMO-responsive UGRO018_00760^{GH4} enzyme after incubation overnight with purified mucin substrates. Respective substrate structures are shown below each condition. **(E.)** A venn diagram showing significantly up-regulated genes when grown in GalNAc, GlcNAc and gMO compared to cellobiose. Significance thresholds include having a fold-change >5-fold with a p-value <0.05. **(F.)** A venn diagram showing significantly down-regulated genes when grown in GalNAc, GlcNAc and gMO compared to cellobiose. Significance thresholds include having a fold-change <5-fold with a p-value <0.05. **(G.)** Gene architecture of a locus containing a YhfC family protease that is upregulated only in the presence of gMO and not GalNAc or GlcNAc when compared to cellobiose. **(H.)** Periodic Acid Schiff (PAS) stained PAGE gel of cultures of *Hm* grown on cMUC2. **(I.)** PAS stained PAGE gel of cultures of *Hm* grown on rich media (chopped meat broth; CMB) containing GlcNAc but not mucin glycoprotein or PGM. Prominent banding in supernatants from *Hm* cells grown in CMB (^) appears to have a similar banding pattern as in PGM (*) lanes implying *Hm* cell-associated proteins are being visualized and the YhfC protease does not demonstrate noticeable PGM glycoprotein degradation. Select molecular weight markers are labeled for panels H., I.

Supplemental Figure 3.

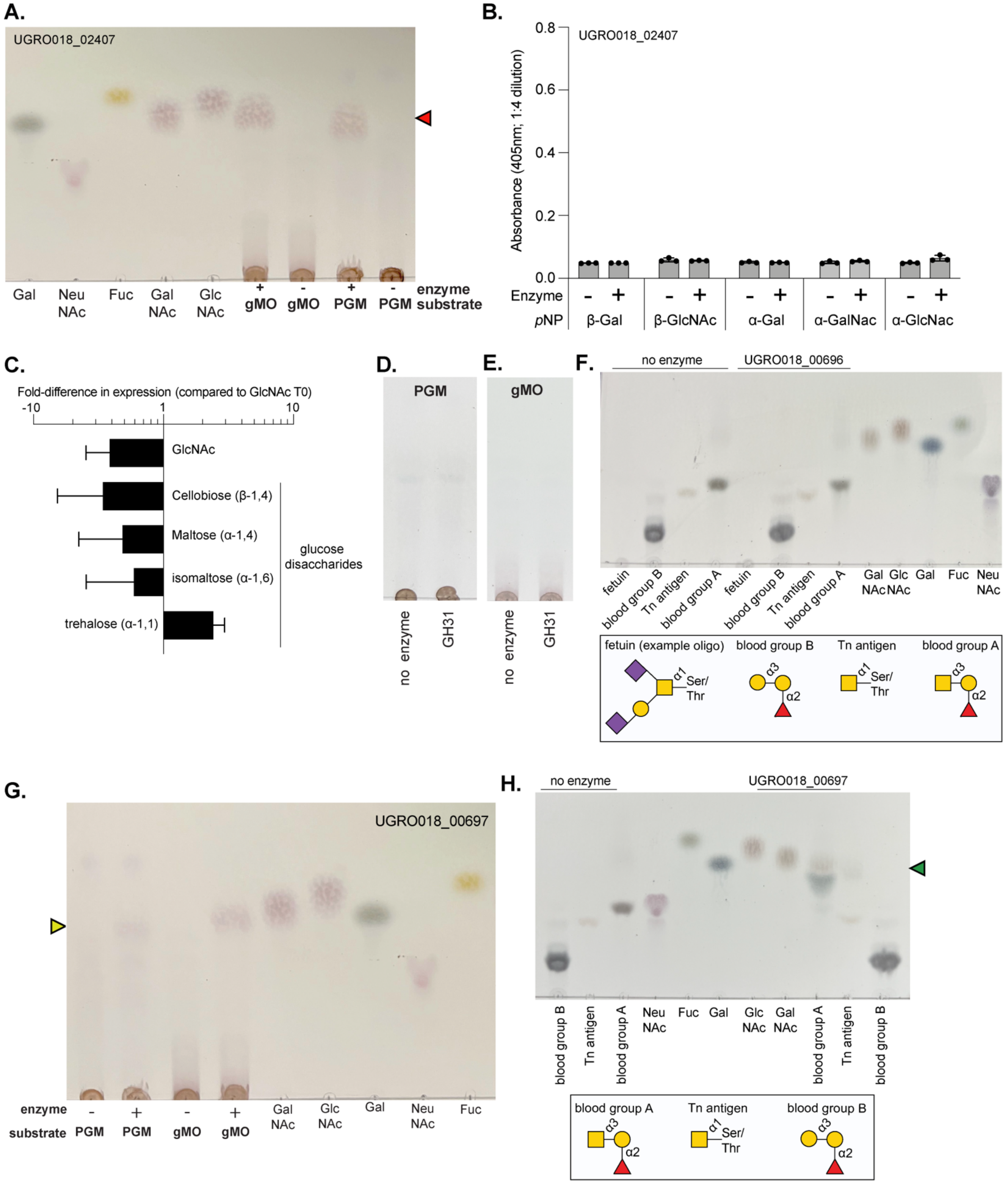

Supplemental Figure 3. (A.) TLC experiment in which recombinantly expressed UGRO018_02407^{GH89} was incubated with gMO and PGM substrates. The red arrow indicates release of GlcNAc in the presence of the enzyme. **(B.)** Endpoint readings at 405nm of overnight incubations with *p*-Nitrophenyl substrates incubated with recombinantly expressed UGRO018_02407^{GH89} enzyme (overnight reactions were diluted 1:4 before reading). **(C.)** Transcript responses by UGRO018_01830^{GH13_29} when grown in GlcNAc to mid-exponential phase before harvesting a subset of cells (Ab_{600} at ~0.6; timepoint 0; T_0) and transferring the remaining cells to medium containing glucose disaccharides (trehalose, cellobiose, maltose, isomaltose; linkages in parentheses) individually, as well as medium containing GlcNAc as a control. After transfer, cells were allowed to incubate for 4hrs before harvesting and measuring UGRO018_01830^{GH13_29} expression. Compared to expression at T_0, all substrates including GlcNAc, exhibited similar UGRO018_01830^{GH13_29} transcript levels apart from trehalose. It does not appear that cellobiose elicits repression of UGRO018_01830^{GH13_29}, at least with respect to GlcNAc and trehalose appears to be an activating signal. Values are the mean ± standard deviation of n=3. **(D.)** TLC of recombinantly expressed UGRO018_00696^{GH31_18} incubated with PGM. **(E.)** TLC of recombinantly expressed UGRO018_00696^{GH31_18} incubated with gMO. **(F.)** TLC of the recombinantly expressed UGRO018_00696^{GH31_18} enzyme after incubation overnight with purified mucin substrates. Respective substrate glycobiological structures are shown below tested conditions. **(G.)** TLC of recombinantly expressed UGRO018_00697^{GH36} incubated with PGM and gMO substrates. The yellow arrow indicates release of GalNAc with the addition of enzyme. **(H.)** TLC of the recombinantly expressed UGRO018_00697^{GH36} enzyme after incubation overnight with purified mucin substrates. Substrate structures are shown below tested conditions. The green arrow indicates release of GalNAc from blood group A and Tn Antigen. Data images shown in panels 3A and 3G are from the same TLC experiment and therefore the three sugar standards (Gal, NeuNAC, Fuc) are from an identical image.

Supplemental Figure 4.

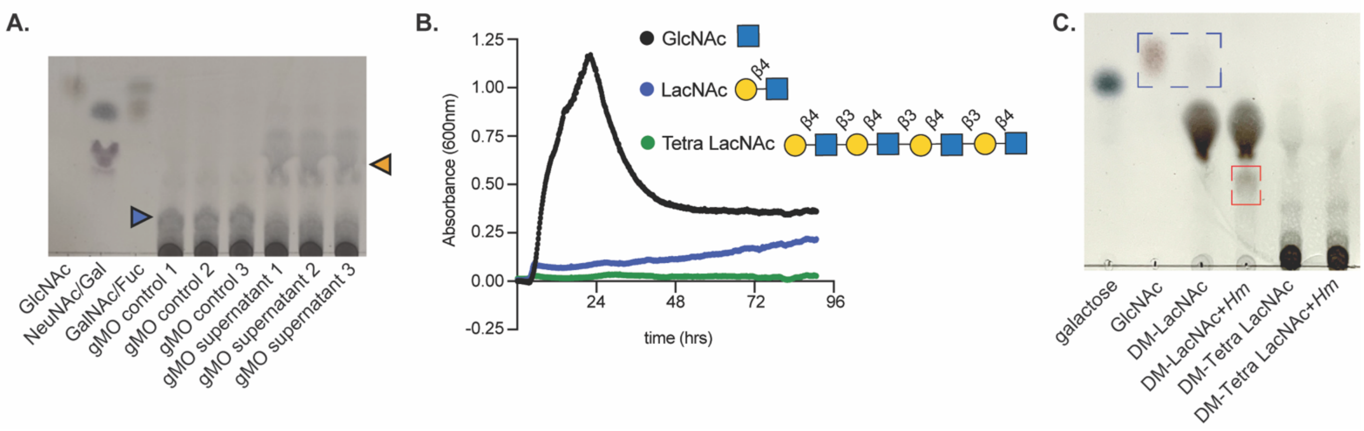

Supplemental Figure 4. (A.) TLC of media containing gMO and the supernatants from gMO-grown *Hm* cultures. The blue arrow indicates the disappearance of higher molecular weight *O*-glycans and the orange arrow indicates appearance of new glycan structures that suggest partial degradation by *Hm*. **(B.)** Growth curves of *Hm* grown on media containing 0.5% GlcNAc, LacNAc and Tetra LacNAc. **(C.)** TLC plate from the culture supernatants in panel B showing no degradation of LacNAc or Tetra LacNAc substrates. The blue box indicates apparent contamination of GlcNAc in the LacNAc, likely responsible for initial growth in the first few hours and the red box indicates the possible precipitate formed causing the increase in optical density after 24 hrs.
